# Drug-Induced Alopecia Areata From Upadacitinib

**DOI:** 10.7759/cureus.66647

**Published:** 2024-08-11

**Authors:** Alexander H Chang, Nicholas D Brownstone, Sylvia Hsu

**Affiliations:** 1 Dermatology, Temple University Hospital, Philadelphia, USA

**Keywords:** rinvoq, alopecia areata (a.a.) treatment, jak inhibitor adverse effects, drug-induced alopecia areata, upadacitinib

## Abstract

This case report describes an unexpected occurrence of alopecia areata (AA) in a 55-year-old woman undergoing treatment with upadacitinib for atopic dermatitis. This patient developed AA approximately one year into her upadacitinib treatment for atopic dermatitis. This case highlights possible upadacitinib-induced AA, which has not been reported in the literature. Not only does this case demonstrate the typical timeline for drug-induced alopecia, but it also raises questions about whether upadacitinib can cause drug-induced alopecia.

## Introduction

Alopecia areata (AA) is an autoimmune disorder characterized by a sudden onset of non-scarring hair loss, which can have a significant psychological impact on patients, affecting their quality of life and emotional well-being [1]. Quite often, the severity of AA is unpredictable, ranging from small patches of hair loss to complete baldness [1]. Recent advancements in AA treatment have led to the exploration of targeted therapies, such as Janus kinase (JAK) inhibitors [2]. JAK inhibitors modulate the JAK-STAT signaling pathway, which is implicated in the pathogenesis of many autoimmune and inflammatory diseases [2]. The US Food and Drug Administration (FDA) has approved two JAK inhibitors, baricitinib and ritlecitinib, specifically for the treatment of AA, underscoring the potential of this therapeutic class in managing the disease [2].

Upadacitinib is a selective JAK1 inhibitor, approved for the treatment of various inflammatory conditions including atopic dermatitis, psoriatic arthritis, Crohn’s disease, rheumatoid arthritis, ankylosing spondylitis, and ulcerative colitis [3]. As a member of the JAK inhibitor class, upadacitinib has demonstrated efficacy in controlling inflammation and modulating immune responses [3]. Although JAK inhibitors are generally well-tolerated, a range of side effects including herpes virus reactivation and nasopharyngitis has been documented [2].

Preliminary reports in the literature suggest that upadacitinib may also be effective in treating AA, with varying timelines for achieving hair regrowth among patients. This emerging evidence underscores the need for further investigation into the role of upadacitinib in managing AA. This case report aims to present and discuss evidence for drug-induced AA potentially caused by upadacitinib.

## Case presentation

**Figure 1:**
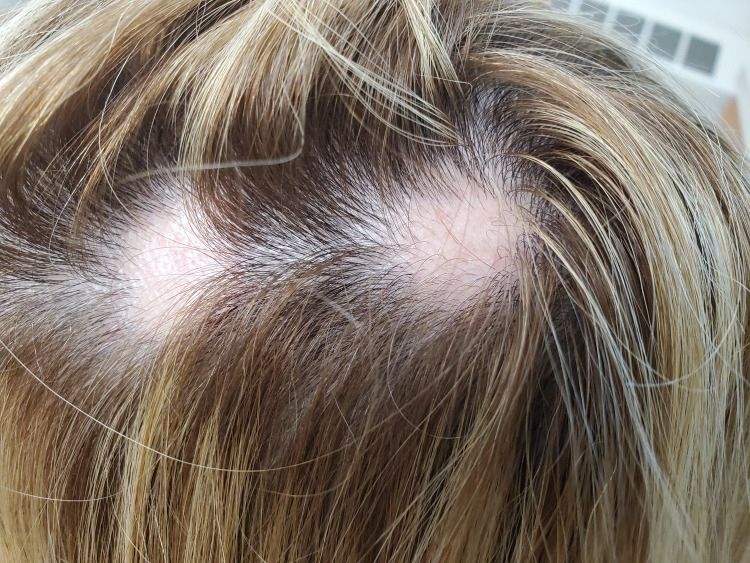
Two round alopecic patches of alopecia areata on the left vertex scalp.

A 55-year-old woman with severe atopic dermatitis was prescribed upadacitinib at an initial dosage of 15 mg daily. After 3 months, the dose was increased to 30 mg daily to achieve better disease control. The patient reported significant improvement in her atopic dermatitis symptoms. However, after nearly one year (11.7 months) of upadacitinib treatment, she noticed two round patches of hair loss on her scalp (Figure 1). She was subsequently treated with intralesional triamcinolone 5 mg/mL injections.

The patient had no prior history of AA. Laboratory investigations, including thyroid function tests and complete blood count, were within normal limits. The patient's treatment regimen will include upadacitinib for her atopic dermatitis and continued intralesional triamcinolone injections for the AA patches.

## Discussion

Alopecia areata is an autoimmune condition, in which the immune system attacks hair follicles resulting in non-scarring hair loss [1]. The paradoxical onset of alopecia areata in a patient undergoing upadacitinib therapy is noteworthy, given that some JAK inhibitors are FDA-approved as treatments for this condition. Upadacitinib inhibits JAK1, and its mechanism has a narrower target than other JAK inhibitors that are FDA-approved for alopecia areata, such as ritlecitinib, a JAK3/TEC-kinase inhibitor, and baricitinib, a JAK1/2 inhibitor [2]. This case raises questions about why alopecia areata would present in a patient undergoing a drug treatment designed to modulate the JAK pathway. The patient, who has no previous history of AA, experienced the onset of AA one year after starting upadacitinib for atopic dermatitis. The one-year latency period is characteristic of other drug-induced cases of AA [4].

The exact mechanism by which upadacitinib can induce AA is not well understood and has not been thoroughly investigated. One possible explanation is that the modulation of JAK1 is not enough - that is, multiple JAK pathways are necessary to be modulated for the prevention of the autoimmune symptoms seen in AA. Alternatively, JAK1 might not be the primary pathway that leads to the emergence or exacerbation of autoimmune conditions. Drug-induced AA typically occurs within one year of starting treatment, suggesting a temporal relationship between the initiation of therapy and the onset of hair loss [4,5].

Based upon a literature review of all AA cases treated with upadacitinib, the timeline of resolution varies widely from 1 month to 10 months (Table 1). There is no convincing evidence for a temporal relationship between the administration of upadacitinib and the improvement of AA. Given the wide range of resolution timelines, it is plausible that the resolution of AA is not linked to upadacitinib, but rather occurs on its own.

**Table 1:** Summary of reported improvement times for alopecia areata patients on upadacitinib Time to improvement is approximate due to the differing methods of reporting and measurement used across various papers; significant improvement was noted as a decrease in Severity of Alopecia Tool (SALT) score over 50.

Authors	Number of Patients	Time to Improvement
Giavina-Bianchi et al. [6]	1	6 months
He et al. [7]	5	3 months (significant improvement in 5 patients)
Muzy [8]	1	4 months (16 weeks)
De la Torre-Gomar et al. [9]	1	5 months
Ha et al. [10]	1	10 months
Novielli et al. [11]	1	1 - 9 months
Uchida et al. [12]	1	4 - 7 months
Walls et al. [13]	1	4 months
Johnston et al. [14]	3	3 to 8 months
Johnston et al. [15]	1	7 months
Kołcz et al. [16]	1	1 month
Youssef et al. [17]	1	4 months
Bourkas et al. [18]	1	6 weeks improvement, whole scalp 5 months
Gori et al. [19]	1	12 weeks
Asfour et al. [20]	1	4 weeks
Cantelli et al. [21]	1	3 months
Gambardella et al. [22]	2	2 months

It has been established that atopic dermatitis increases the risk of developing AA, which would suggest that the patient's AA emerged as part of an autoimmune complication [23]. However, the one-year period from the initial administration of upadacitinib to the onset of AA, which coincides with the typical timeline for drug-induced AA, combined with the paradoxical occurrence of AA while under a treatment intended to treat it, challenges the assumption that the patient's AA is solely related to her underlying atopic dermatitis and poses the possibility of drug-induced AA from upadacitinib.

Understanding the complex relationship between JAK inhibition and autoimmune responses warrants further investigation. Investigating the differences between upadacitinib and other JAK inhibitors that target multiple JAK pathways could provide insights into why AA can present in a patient on upadacitinib. Further research is needed to determine whether AA is a rare adverse effect of upadacitinib or if JAK1 inhibition alone is inadequate for immune modulation. Additionally, understanding patient-specific traits and the determinants of immune modulation in paradoxical presentations of AA could help guide future research into JAK inhibitors.

## Conclusions

This is the first known case in the literature of upadacitinib-induced AA. Case reports in the literature have postulated that upadacitinib may be a treatment for AA, but the timeline to improvement widely varies with no convincing temporal evidence to establish a link between upadacitinib and the resolution of AA. While upadacitinib is effective in managing atopic dermatitis, clinicians should be aware of the potential for rare adverse effects, such as AA. Further research is needed to better understand this rare presentation, including the possibility that inhibition of JAK1 alone may not be sufficient, potentially leading to drug-induced alopecia areata.
